# Phosphorylation at Serines 216 and 221 Is Important for *Drosophila HeT-A* Gag Protein Stability

**DOI:** 10.1371/journal.pone.0075381

**Published:** 2013-09-18

**Authors:** Sukhdev S. Brar, Robert M. Petrovich, Jason G. Williams, James M. Mason

**Affiliations:** 1 Laboratory of Molecular Genetics, National Institute of Environmental Health Sciences, National Institutes of Health, Research Triangle Park, North Carolina, United States of America; 2 Laboratory of Structural Biology, National Institute of Environmental Health Sciences, National Institutes of Health, Research Triangle Park, North Carolina, United States of America; University of Medicine and Dentistry of New Jersey, United States of America

## Abstract

Telomeres from *Drosophila* appear to be very different from those of other organisms – in size and the mechanism of their maintenance. In the absence of the enzyme telomerase, *Drosophila* telomeres are maintained by retrotransposition of three elements, *HeT-A*, *TART*, and *TAHRE*, but details of their transposition mechanisms are not known. Here we characterized some biochemical characteristics of the *HeT-A* Gag protein encoded by the *HeT-A* element to understand this mechanism. The *HeT-A* Gag protein when overexpressed in S2 cells was localized to the nucleus but was resistant to high salt, detergents and nuclease extraction treatments. Analysis of the *HeT-A* Gag protein by tandem mass spectrophotometry revealed that serines 216 and 221 are phosphorylated. Substituting these serines with alanine or aspartic acid by site-directed mutagenesis did not result in any changes in *HeT-A* Gag translocation across the nucleus, suggesting that phosphorylation of these sites is not associated with *HeT-A* Gag translocation, but time course experiments showed that these phosphorylation sites are important for Gag-protein stability.

## Introduction

Telomeres are nucleoprotein complexes that are associated with the ends of chromosomes. Telomere maintenance is a complex process and is essential for cell replication and genome integrity. Cells that have shortened telomeres enter senescence faster than normal cells. Appropriate telomere length in plants, animals, protozoans, and fungi is maintained primarily by the enzyme telomerase. *Drosophila* lacks this enzyme and has a different system of maintaining telomere length. Telomeres in *Drosophila* are maintained using three non-long terminal repeat (LTR) retrotransposable elements, *HeT-A*, *TART*, and *TAHRE* (jointly abbreviated HTT), which only attach at the ends of the chromosomes [1], [2], [3]. It has also been shown that, in addition to transposition, elongation of the HTT array can also occur by an alternate mechanism, such as gene conversion/recombination [4].

Non-LTR retrotransposons generally carry a gag and a pol gene and have an oligo(A) tract at the 3′ end [1], [5], [6], [7]. *HeT-A* in *Drosophila* encodes only for a Gag-like protein while *TART* and *TAHRE* encode both a Gag-like protein and a reverse transcriptase (pol gene product). A unique characteristic of the HTT elements is that they transpose only to ends of chromosomes. These three elements transpose independently of each other and are arranged in tandem repeats. The exact mechanisms of transposition of these elements, their interactions with other proteins or any posttranslational modifications they undergo are still obscure. Our current understanding of *HeT-A* (or *TART*) transposition comes by analogy with other retroelements and retroviruses [8]. In HIV-1, for example, Gag/Gag, Gag/Gag-Pol dimerization and higher-order multimerization events and also Gag phosphorylation are known to occur during the HIV-1 life cycle [9], [10], [11]. Based on structural similarity between the HIV-1 and the *HeT-A* Gag proteins, it is speculated that the *HeT-A* protein interacts with itself and with its mRNA to form a larger complex in the cytoplasm which then translocates into the nucleus and eventually to the telomeres. It is further possible that this large complex may interact with other proteins (possibly telomere cap proteins) before it reaches the telomeres. It is reasonable to ask whether, during its journey from the cytoplasm to the telomeres, the *HeT-A* Gag protein may undergo some kind of post-translational modifications which may be necessary for its interaction with other proteins or for targeting to a specific site.

Protein phosphorylation is known to play an important role in protein-protein interactions [12], [13], translocation of certain proteins to the nucleus [14], [15] and generating the physiologically active form of some proteins [16], [17]. In most retroviruses the Gag proteins are subjected to many modifications both during and after their synthesis. For example, in HIV-1 Gag undergoes amino-terminal modification by the addition of myristate that is required for the binding of Gag to the plasma membrane [18],[19],[20]. The HIV-1 Gag also undergoes phosphorylation that is associated with the integration of complex during viral entry [21], [22], [23].

Our current understanding of how *HeT-A* Gag in *Drosophila* is transposed to chromosome ends and its involvement in telomere elongation is limited by our lack of knowledge of its structural and biochemical characteristics during and after synthesis. To identify some of its protein characteristics or post-translational modifications, if any, we expressed a FLAG-tagged construct of *HeT-A* Gag in S2 cells and analyzed this recombinant *HeT-A* Gag protein by tandem mass-spectrometry. Using this methodology we identified two serine sites that are phosphorylated. We then asked whether phosphorylation at these sites is important for *HeT-A* Gag translocation into the nucleus or its stability. We also applied various extraction methodologies, such as high salt, nuclease and detergents, in an attempt to extract *HeT-A* Gag expressed in stable cell lines and to study any possible interaction with other cellular proteins.

## Materials and Methods

### Reagents

Electrophoretic-grade reagents: Sodium chloride, sodium deoxycholate, sodium dodecyl sulfate (SDS), Tween-20, Tris, and non-fat dry milk powder were obtained from Biorad laboratories (Hercules, CA). Nitrocellulose membranes were obtained from Amersham Biosciences (Piscataway, NJ). Proteasome inhibitor MG132 and 3x FLAG antibody were from Sigma (St. Louis, MO), HP1 from Active Motif (Carlsbad, CA) and actin was purchased from Santa Cruz Biotechnology (Santa Cruz, CA). The electrochemiluminescence (ECL) western blotting kit including horse-radish peroxidase-conjugated secondary antibodies were obtained from Amersham Bioscience (Piscataway, NJ). Unless otherwise stated, cell culture reagents were from Invitrogen (Carlsbad, CA) and all biochemical reagents used were from Sigma Aldrich, Inc. (St. Louis, MO).

### Cell Culture

Drosophila S2 cells were grown at 25°C in Schneider's Drosophila Medium supplemented with 10% heat-inactivated fetal bovine serum (Invitrogen, Carlsbad, CA) and 1x antibiotic-antimycotic (Invitrogen).

### Cloning in PMK33 Vector for making stable S2 cell lines

The vector PMK33-CFH-BD (8550 bp) was kindly provided by Kenneth H. Wan (Berkeley Drosophila Genome Project, Lawrence Berkeley National Laboratory, CA). This vector was modified by inserting a 3x FLAG tag at the BamH1 site (6927–6933). Briefly, a 99 bp 3x FLAG tag sequence with BamH1 sequence at both ends and a Sma1 site at the 5′ end was synthesized by GenScript (Piscataway, NJ) and cloned into the pUC57 vector. The 3x FLAG tag was released from pUC57 by BamH1 restriction digest and cloned into the BamH1 site in PMK33-CFH-BD. Next, the 2.8 kb *HeT-A* ORF1 was amplified by PCR from the 9D4 Y.E.S. plasmid (kindly provided by Dr. Pam Geyer, University of Iowa), with primer pairs 9D4-F8 and 9D4-R8 (Table 1) and cloned into the Sma1 site by blunt end ligation. The modified PMK33-CFH-BD vector was transfected into S2 cells using the Amaxa Cell Line Nucleofactor Kit V as per the manufacturer's instruction. Cells were incubated for 2 days in Schneider S2 media followed by selection with 300 µg/ml of Hygromycin B (Invitrogen). Stable cell lines expressing FLAG tagged *HeT-A* Gag were confirmed by PCR using a vector specific primer (PMK33-1) and an insert specific primer (9D4 R4) (Table 1) and by immunoblot using anti-FLAG antibody.

**Table 1:** Primers for PCR, sequencing, and site-directed mutagenesis.

Primer	Sequence
9D4-F8	CTGGAGGACACATTACAATTAAAAAGC
9D4-R8	ATTGGATGTATTCATGTCCAGATTGTTATTTC
9D4 R4	CCTCATATGCGTGTGCGGTGGACGGAGGAG
pMK33-1	TGCACACGTCTCCACTCGAATTTG
pMK33-2	AATCGAACGAAAGACCCGTGTGTAAAG
pMK33-3	ATTCAAATATGTATCCGCTCATGAGAC
pMK33-4	GAAGCTTGAGCTCGAGATCCACGTCAG
GAPDH-5	TTTCTCAGCCATCACAGTCG
GAPDH-6	CGACCTCCTCATCGGTGTAT
SDMGag-1	AAAGCCAATGTTAATGACGCTGGGGAAATATTCTCCCCAC
SDMGag-2	GTGGGGAGAATATTTCCCCAGCGTCATTAACATTGGCTTT
SDMGag-3	GACGCTGGGGAAATATTCGCCCCACTTATACAAATTGACG
SDMGag-4	CGTCAATTTGTATAAGTGGGGCGAATATTTCCCCAGCGTC
SDMGag-9	AAAGCCAATGTTAATGACGATGGGGAAATATTCGACCCACTTATACAAATTGACG
SDMGag-10	CGTCAATTTGTATAAGTGGGTCGAATATTTCCCCATCGTCATTAACATTGGCTTT

### Mutant *HeT-A* Gag

Mutant versions of *HeT-A* Gag (M1, M2, and M3) were made by site-directed mutagenesis using the STRATAGENE kit (La Jolla, CA) as per the manufacturer's instructions. Primers and templates used for site-directed mutagenesis are shown in Table 2. Stable S2 cell lines expressing wild type or mutant *HeT-A* Gag proteins were confirmed by immunoblot using anti-FLAG antibody, after induction with CuSO_4_.

**Table 2:** Mutagenesis sites, primers and templates for site-directed mutagenesis.

Stable Cell Line	Location 216	Location 221	Primers	Template
Wild Type	Serine (AGT)	Serine (TCC)		
M1-mutant	Alanine (GCT)	Serine (TCC)	SDMGag 1 and 2	Wild Gag
M2-mutant	Alanine (GCT)	Alanine (GCC)	SDMGag 3 and 4	Gag-M1
M3-mutant	Aspartic (GAT)	Aspartic (GAC)	SDMGag 9 and 10	Gag-M2

### Confocal microscopy

For *HeT-A* Gag-FLAG detection by confocal microscopy, stably expressing *HeT-A* Gag-FLAG S2 cells were induced with 500 µM CuSO_4_ for 48 hrs. Cells were collected by centrifugation and dropped onto *Superfrost/Plus* microscope slides (Fisher Scientific, Pittsburg, PA). Cells were allowed to settle for 10 min and then fixed with 4% paraformaldehyde in PBS for 15 min, washed once and permeabilized with 0.2% Triton-X100 in PBS for 5 min. The cells were washed three times for 5 min with PBS and blocked with blocking buffer (2% BSA-PBS) for 1 hr. After washing once with PBS, cells were incubated overnight at 4°C with alexa fluor 555 conjugated anti-FLAG M2 antibody (Cell Signaling, Danvers, MA) diluted in 1% BSA-PBS. The slides were rinsed three times in PBS for 5 minutes each, dried and coverslips mounted with Prolong Gold Antifade Reagent (Invitrogen). Images were collected using a Zeiss LSM 510 laser scanning confocal microscope equipped with 60× objective.

### Immunoblot Analysis

Stable S2 cell lines expressing *HeT-A* Gag-FLAG were induced with 500 µM CuSO_4_ for 48 hrs. Cells were collected by centrifugation and lysed in Laemmli Sample Buffer (Bio-Rad, Hercules, CA) in the presence of Halt Protease Inhibitor Cocktail (Thermo Scientific, Rockford, IL) and lysate broken by sonication. Cell lysates were subjected to electrophoresis in 4–12% SDS-polyacrylamide gels (Invitrogen) and then transferred to nitrocellulose membranes. After blocking with 5% dry milk in PBST, membranes were probed with anti-FLAG at 1∶5000 dilution for 1 hr at room temperature followed by species-specific, secondary antibody. Immunoblots were detected by enhanced chemiluminescence method. Membranes were stripped and probed for actin and histone H1 antibodies as loading controls. Cytosolic and nuclear fractions used in immunoblot analysis were prepared by using NE-PER Nuclear and Cytoplasmic Extraction Kit (Pierce, Rockford, IL) as per manufacturer's instructions.

### RT-PCR

Total RNA was harvested from normal S2 and *HeT-A* Gag expressing stable cell lines using RNeasy Mini Kit (QIAGEN, Valencia, CA) according to the manufacturer's instructions. First-strand cDNA was synthesized from 2 µg of total RNA using random hexamer (Roche, Applied Biosystems, Branchburg, NJ) and Superscript II RNase H Reverse Transcriptase (Invitrogen) and PCR was performed using gene specific primers (Table 1).

### Cytological Preparations

S2 stable cell lines expressing wild or mutant *HeT-A* Gag-FLAG were induced with 500 µM CuSO_4_ for 48 hrs and arrested with colcemid at a concentration of 0.1 µg/ml for 2 hrs. Pelleted cells were washed in PBS, spun down and re-suspended in 5 ml of 0.5% (w/v) sodium citrate (hypotonic solution) for 10 min. Cells were spun, and resuspended in a small volume of hypotonic solution left after the removal of supernatant. 50 ul of this suspension was placed in a single-chamber Cytospin funnel and spun for 5 min at 900 rpm (high acceleration) in a Shandon Cytospin 3. The cells were fixed with 4% paraformaldehyde for 10 min, washed with PBS, and permeabilized with 0.1% Triton X-100 for 5 min. After washing with PBS, cells were blocked with 2% BSA-PBS for 1 hr and stained with alexa fluor 555 conjugated anti-FLAG M2 antibody (Cell Signaling) as mentioned above in the confocal microscopy section.

### Detergents, DNase/RNase, Benzonase and Proteinase K treatments

S2 stable cells expressing wild *HeT-A* Gag-FLAG were induced with 500 µM CuSO_4_ for 48 hrs. Cells were spun and cytoplasmic/nuclear/nuclei fractions were prepared by using NE-PER Nuclear and Cytoplasmic Extraction Kit (Pierce) as per the manufacturer's instructions. For detergent experiment, nuclei were treated with 0.5 M NaCl, 1.0% Triton-X100 and 40 mM CHAPS for 30 minutes. SDS treatment of nuclei was performed at room temperature for 10 min. Supernatant (S) and pellet (P) fractions were separated by centrifugation for 10 min at 13,000 rpm at 4°C and subjected to immunobot analysis. For DNase/RNase treatment, 50 ul of nuclear extract was separately treated with 15,000 units of DNase (QIAGEN) or 100 µg of RNase-A or with both DNase and RNase-A for 30 min at room temperature. Before separating supernatant and pellet fractions, a 10 ul aliquot was removed to run on an agarose gel to verify completion of the reaction; the remaining fraction spun as mentioned above to separate S and P fractions for immunoblot analysis. For the benzonase nuclease experiment, nuclei were treated with 500 units of benzonase for 30 min, 1, or 2 hrs at room temperature and samples processed as performed after DNase/RNase treatment and subjected to immunoblot analysis. For the proteinase-K experiment, nuclei (50 µl nucleoprotein mixture - well mixed) were treated with various concentrations (0, 1, 5, or 10 µg proteinase-K) for 30 minutes at room temperature. Proteinase-K treatment was also performed at room temperature for various time points (0, 1, 5, and 30 min) in a 50 µl nuclear fraction containing 5 µg of proteinase-K. Reactions were spun as above to separate S and P fractions and subjected to immunoblot.

### Cyclosporine-A treatment

Stable S2 cell lines expressing wild *HeT-A* Gag-FLAG were treated with various concentration (1, 5, or 10 µM) of cyclosporine-A for 4 hrs then induced with CuSO_4_ for 48 hrs. Cells were collected by centrifugation, and cytoplasmic/nuclear/nuclei fractions were prepared by using NE-PER Nuclear and Cytoplasmic Extraction Kit (Pierce) as per the manufacturer's instructions. Nuclei were resuspended in TE buffer and broken with one or two control bursts from a sonicator (Microson, Farmingdale, NY). Cytoplasmic, nuclear, and nuclei fractions were subjected to immunobot and probed with anti-FLAG antibody. The same membrane was stripped and probed with actin and histone H1 antibodies as loading controls for cytoplasmic and nuclear fractions respectively.

### Mass Spectrometry Analysis

In-gel digestion and mass spectrometry where performed essentially as described previously. [24].

## Results

### Recombinant *HeT-A* Gag expressed in S2 cells is localized to the nucleus

**Figure 1:**
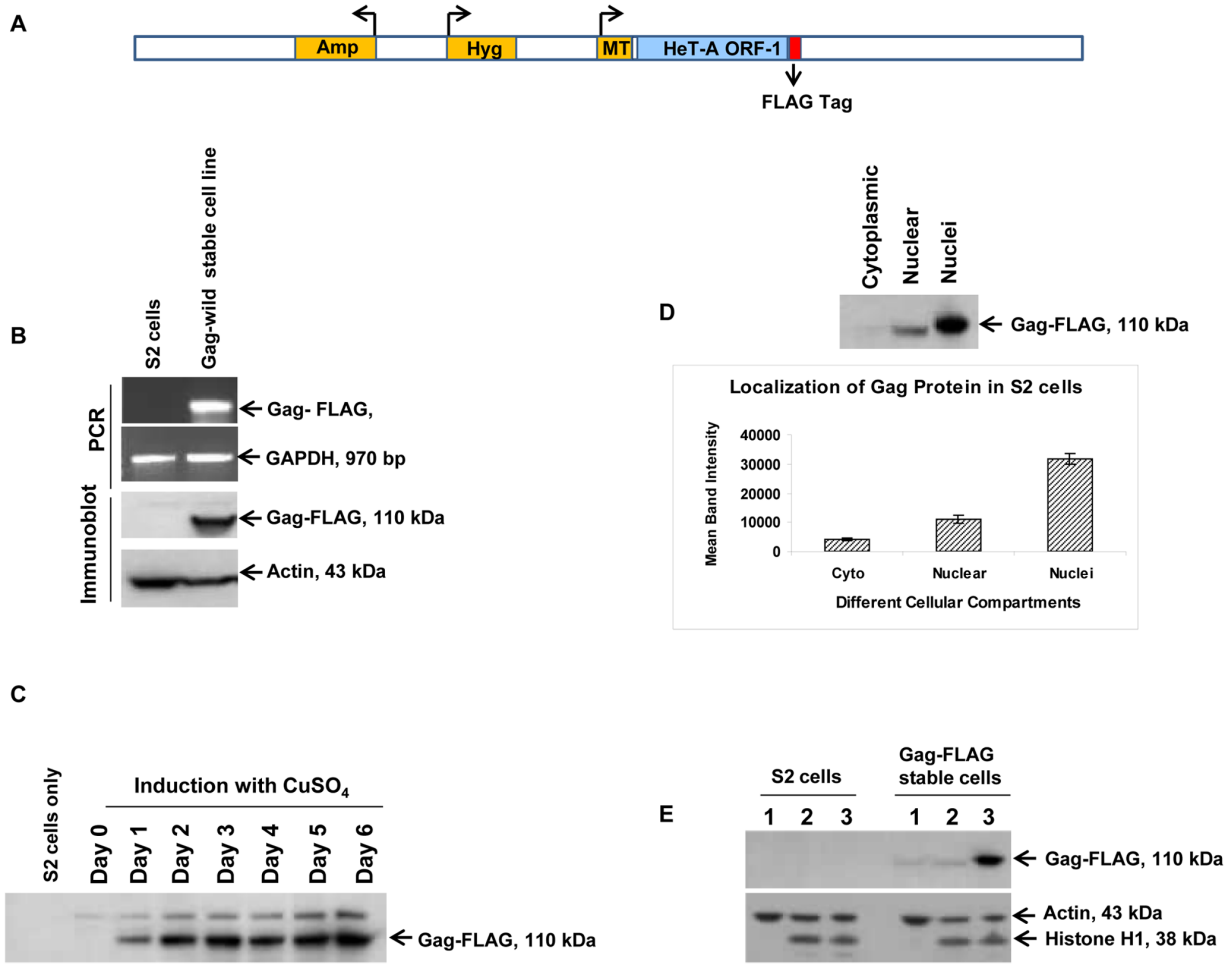
Making of a *HeT-A* Gag-FLAG stable cell line. (A) Line diagram of the PMK33 vector showing important genetic markers with *HeT-A* Gag ORF1 cloned at the SmaI restriction site and a 3x FLAG tag at the C-terminus. (B) Expression of *HeT-A* Gag-FLAG in S2 cell lines as analyzed by PCR and immunoblot. GAPDH and actin serve as internal controls for PCR and immunoblots respectively. (C) Stability of expressed *HeT-A* Gag-FLAG protein in S2 cells. Cells were seeded in a T-25 flask and induced with 500 µM CuSO4. 1.0 ml cells were collected each day for a total of 6 days and analyzed by immunoblotting. (D) *HeT-A* Gag-FLAG is targeted to the nucleus. Stably transfected S2 cells were induced with CuSO_4_ and 48 hr post-induction cytoplasmic and nuclear fractions were analyzed by immunoblot. (E) *HeT-A* Gag-FLAG is only detected in stably transfected S2 cells and not in S2 cells. Cytoplasmic, nuclear, and nuclei fractions from both S2 and stably transfected S2 cells were analyzed by immunoblot (1 =  cytosolic, 2 =  nuclear, and 3 =  nuclei).

The *HeT-A* Gag protein when over-expressed in *Drosophila* Schneider line 2 (S2) has been shown to be rapidly transported into the nucleus [25], [26]. We tagged *HeT-A* Gag protein with a FLAG-tag (Fig. 1A) and made a stable S2 cell line. Cells induced with 500 µM CuSO_4_ for 48 hrs showed expression of full length recombinant *HeT-A* Gag protein that was confirmed by immunoblotting (Fig. 1B). Accumulation of recombinant *HeT-A* Gag protein in the cells increased over a period of time and was detected up to day 6 by immunoblotting (Fig. 1C). Immunoblotting of different cellular fractions showed that the recombinant *HeT-A* Gag protein was mostly localized in the nuclear fraction (Fig. 1D, E).

### Serines 216 and 221 in *HeT-A* Gag are phosphorylated

**Figure 2:**
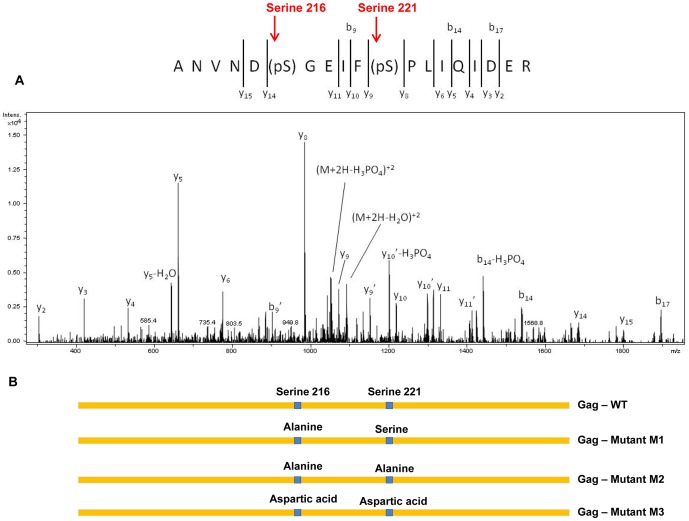
Tandem MS analysis of *HeT-A* Gag protein by mass spectrophometry. (A) MS/MS of ion m/z shows an extensive y-ion series and the presence of phosphorylation at serines 216 or 221, as indicated by the arrow. (B) Line diagram of different forms of *HeT-A* Gags (M1, M2, and M3) that were generated by site directed mutagenesis. Primer sets and template used for site directed mutagenesis are described in Table 2.

In higher eukaryotes phosphorylation occurs on serine, threonine and tyrosine residues, and these phosphorylations are essential for many biological processes [27]. In order to investigate if such phosphorylation events are taking place on the *HeT-A* Gag protein, nuclear extracts from a stable S2 cell line expressing wild *HeT-A* Gag-FLAG tagged protein were analyzed by tandem mass spectrometry. This analysis revealed that serines 216 and 221 could both be phosphorylated (Fig. 2A). This MS/MS spectrum of ion m/z 733 has an extensive y-ion series and based upon the precursor mass clearly contains a single site of phosphorylation. Interestingly, this spectrum appears to be chimeric and has contributions from two co-eluting species, one in which phosphorylation is on serine 216 and a second species in which phosphorylation is on serine 221. There is weak evidence that both serine 216 and serine 221 can be simultaneously phosphorylated, as a low-abundance ion at m/z 760 is observed. This ion nearly co-elutes with the singly phosphorylated species at m/z 733, but MS/MS data were not obtained.

### Wild and mutant *HeT-A* Gag-FLAG proteins in S2 cells are localized to the nucleus

**Figure 3:**
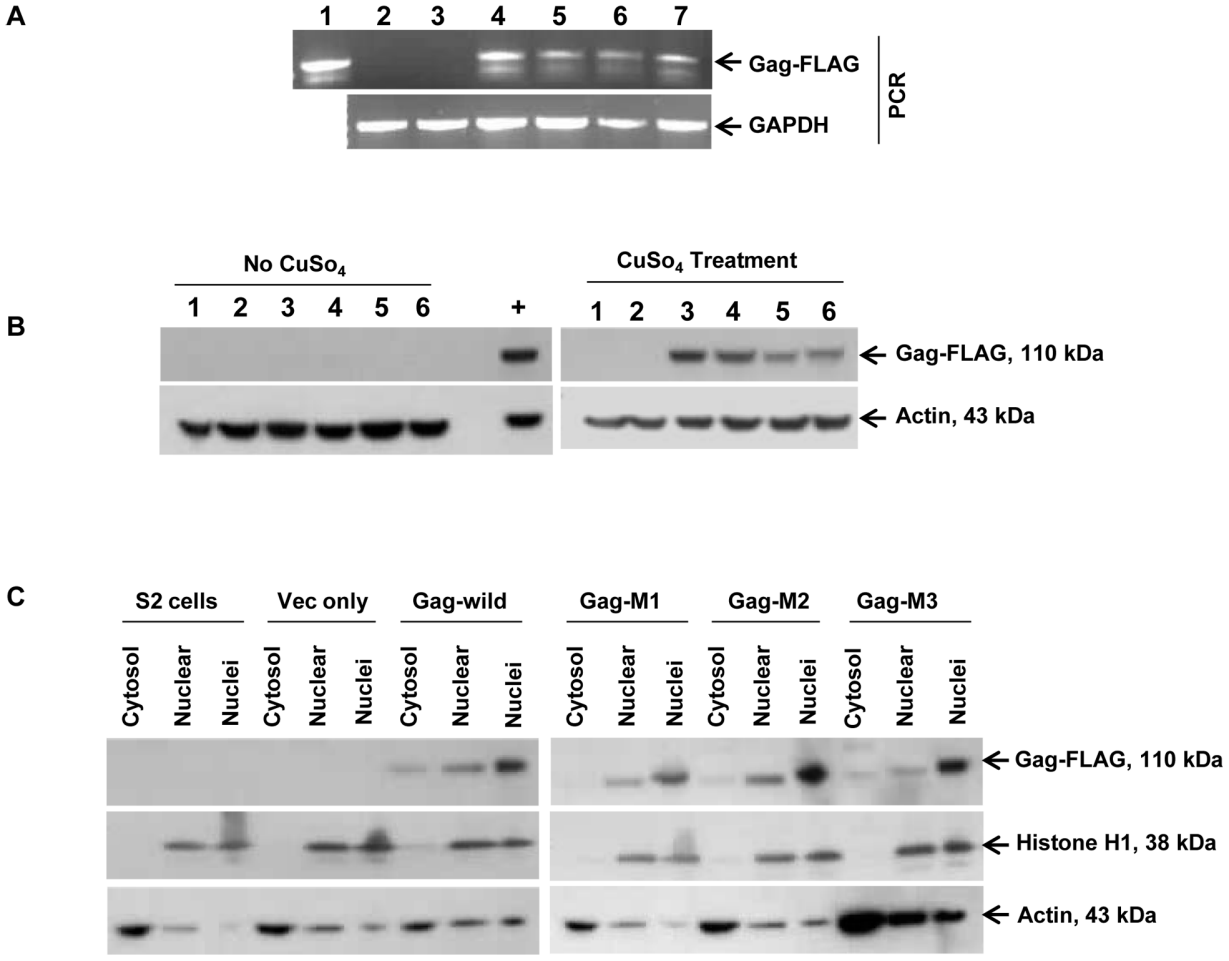
Confirmation of stable cell lines expressing *HeT-A* Gag proteins. (A) The stable cell lines were confirmed by isolating genomic DNA from cells and using a vector specific primer (PMK33-1) and an insert-specific primer (9D4 R4) by PCR. 10 ul of PCR product was run on 1% agarose gel and stained with ethidium bromide and photographed (Lane 1 =  Pos, Lane 2 = S2 cells only, Lane 3 =  Vector alone, Lane 4 =  wild type *HeT-A* Gag, Lane 5 = M1 Gag, Lane 6 = M2 Gag, Lane 7 = M3 Gag). (B) Wild type and mutant *HeT-A* Gag stable cell lines expressed the same size Gag proteins as confirmed by immunoblot. Expression was only seen when cells were induced with CuSO_4_ (lane 1 = S2 cell, Lane 2 =  Vector alone, Lane 3 =  wild type Gag, Lane 4 = M1 Gag, Lane 5 = M2 Gag, Lane 6 = M3 Gag. (C) In wild type and mutant stable cell lines Gag protein was localized in the nuclear fraction as confirmed by immunoblot.

**Figure 4:**
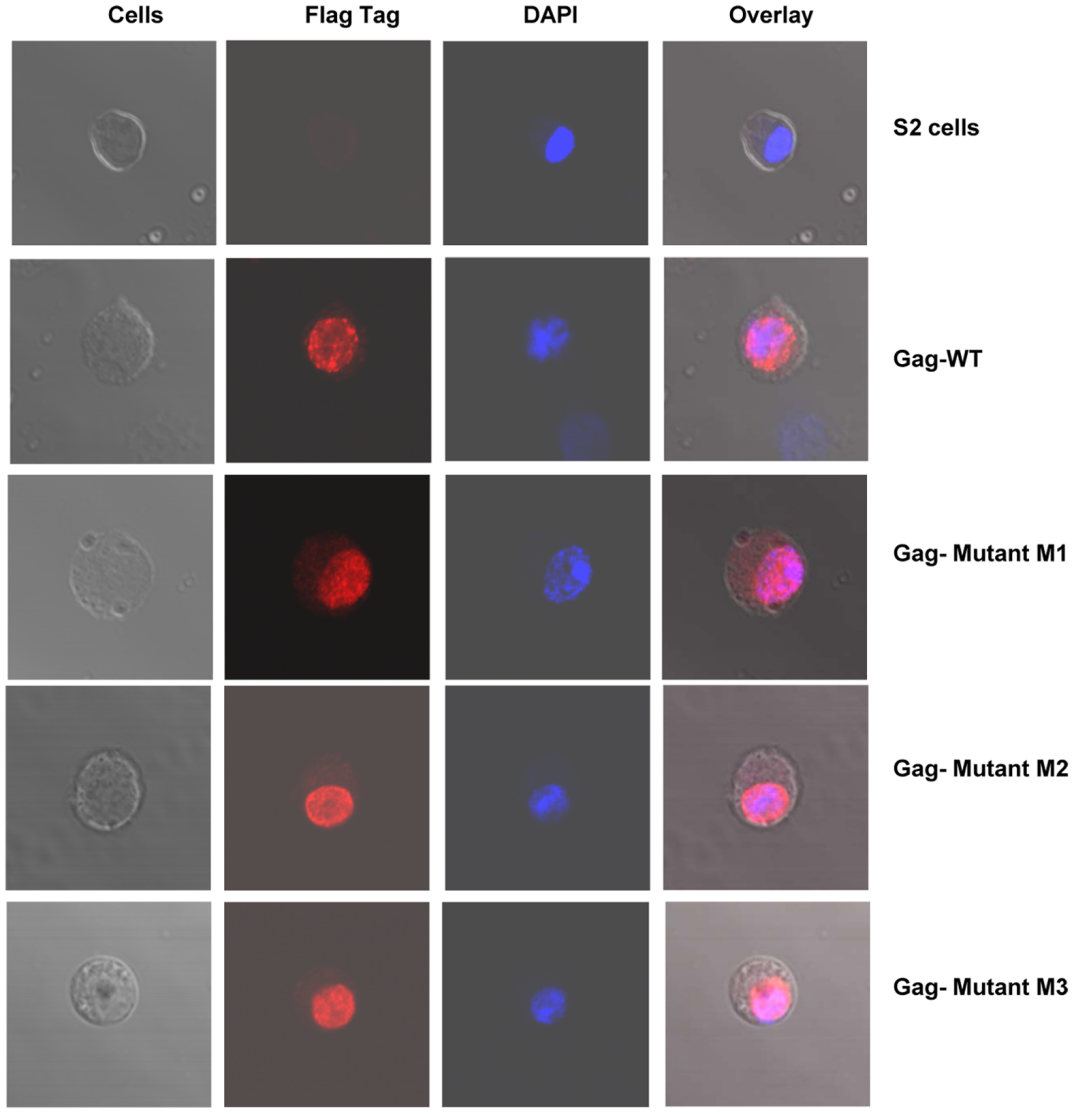
Localization of *HeT-A* Gag-FLAG proteins in S2 cells by confocal microscopy. Stable S2 cells expressing wild and mutant (M1, M2, and M3) *HeT-A* Gag-FLAG protein were induced with CuSO_4_, plated on a cover slip and stained with anti-FLAG antibody. Untransfected S2 cells were used as control. In all four cells lines tested *HeT-A* Gag was localized to the nucleus.

When the *HeT-A* Gag protein is expressed in S2 cells it is rapidly transported into the nucleus [25], [26]. We substituted these two serines by site-directed mutagenesis and generated mutant *HeT-A* Gag M1 (S216A), M2 (S216A, S221A) and M3 (S216D, S221D). Stable cell lines expressing mutant Gags were made as described for wild type *HeT-A* Gag and were induced with CuSO_4_, Wild and mutant *HeT-A* Gag expressing stable cell lines when induced with CuSO_4_ expressed the same size protein (Fig. 3B) and they all localized to the nucleus as confirmed by immunoblot (Fig. 3C). To confirm this finding by confocal microscopy, stable cells were plated on a cover slip and stained with anti-FLAG antibody and viewed under a confocal microscope. Non-transfected S2 cells were used as control. In all four cell lines tested (wild type, M1, M2, and M3) HeT-A Gag was localized to nucleus (Fig. 4). In the wild type and M1 mutant cells the label was found in a punctate pattern, as seen previously [25], [26], while in the M2 and M3 cells the label seemed to be more evenly distributed over the nucleus.

### Recombinant *HeT-A* Gag remains bound to an insoluble nuclear fraction under conditions of high salt, detergent, DNase, RNase, and benzonase nuclease treatment

A number of proteins are known to be tightly bound to DNA; they are referred to as tightly bound proteins (TBPs). These proteins can bind to DNA either through covalent or noncovalent interactions and, even when noncovalently bound, can sometimes withstand high salt and detergent conditions [28]. The wild type *HeT-A* Gag-FLAG protein when expressed in S2 cells is mostly found in an insoluble nuclear fraction (Fig. 1D). Interestingly, *HeT-A* Gag-FLAG is not extractable from this insoluble fractions by either high salt or detergent, such as 0.5 M NaCl, 1% Triton X-100, 40 mM CHAPS, NaCl (0.5–2.0 M), and SDS (0.01–2%) (Fig. S1). This interaction was not disrupted even at 6% SDS (data not shown). The only apparent treatment that releases intact *HeT-A* Gag-FLAG protein from this insoluble pellet is boiling the sample under reducing conditions in 2% SDS. Treatment of cellular nuclei with DNase, RNase, DNase+RNase, benzonase nuclease, or benzonase nuclease plus salt and detergent did not release *HeT-A* Gag protein into solution, which remained in the pellet fraction as seen by immunoblotting (Fig. S1), suggesting that the recombinant *HeT-A* Gag protein was not associated exclusively with either DNA or RNA inside the nucleus. This is not surprising, since a number of other tightly bound proteins, resistant to treatment to nuclease, salts and detergents, are known to be part of the nuclear matrix and are involved in DNA replication, transcription, repair or recombination [28], [29].

### Proteinase K treatment of nuclei resulted in rapid loss of recombinant *HeT-A* Gag protein

Proteinase K is a nonspecific serine protease that preferentially digests proteins after hydrophobic amino acids and rapidly inactivates nucleases. Initial studies on the *HeT-A* Gag protein [30] have shown by northern blot that the association between *HeT*-*A* RNA and Gag protein is very tight, and this association can only be disrupted by Proteinase K and SDS treatment. Such associations are not common, but a few proteins are known that are tightly bound to the nuclear matrix. These proteins fall under the category of TBPs and can only be isolated with DNase and RNase treatment, followed by extractions with high salt [28]. TBPs include diverse class of proteins such as transcription factors, protein kinases, serpins, and proteins of retrotransposons [31]. When we failed to extract recombinant *HeT-A* Gag from nuclei using conventional extraction methods, we thought that the Gag protein may be a TBP. We therefore tried multiple extraction methods with *HeT-A* Gag but none of those methods successfully solubilized *HeT-A* Gag. However, when the nuclear extract was treated with various concentrations of Proteinase K, the recombinant *HeT-A* Gag-protein was completely digested (Fig. 1A), in fact Proteinase K rapidly proteolyzed recombinant *HeT-A* Gag within 1 minute of treatment (Fig. 1B). This suggests that the *HeT-A* Gag protein and/or Gag protein complex is soluble and available for proteolitic digestion and not trapped inside a membrane, protected by a membrane layer or extensively aggregated.

### Cyclosporine-A treatment did not block Gag translocation to nucleus

The Gag protein from HIV-1 has been shown [32] to bind to Cyclophilins A and B, and this interaction is inhibited by cyclosporine-A. Cyclophilins (CyPs) are proteins that catalyze the isomerization of prolines. In the human genome, 17 different isoforms of CyPs are known to exist [33]. The CyPs were originally identified as cellular proteins that bind the immunosuppressive drug cyclosporine A (CsA) [34] but now have been implicated in diverse signaling pathways such as mitochondrial apoptosis [35], [36], RNA splicing [37], [38], and adaptive immunity [39]. In addition, this protein may have a role in directing the proper folding of cellular proteins [40], [41], [42] and directing proteins to different locations within cells [43]. Luban et al. [32] have shown that the HIV-1 Gag protein binds CyPs A and B, and this binding persists under conditions of high salt and detergent but is disrupted by 0.8 µM cyclosporine A. To investigate if the *HeT-A* Gag protein interacts with CyPs, we treated stable cell lines expressing wild type *HeT-A* Gag-FLAG with various concentrations of cyclosporine-A. We hypothesized that because Gag proteins do not have a nuclear localization sequence (NLS), that their sub-cellular localization may be directed by interactions with other proteins. Because cyclophilins are known to direct different proteins to different locations within cells, we performed Western blot analysis of different cellular fractions – cytoplasmic, nuclear, and nuclei from untreated and cyclosporine-A treated samples. These experiments indicated that *HeT-A* Gag protein was mostly present in the nuclear fraction (Fig. S2) and no changes in the *gag* expression profile was seen even at 100 µM cyclosporine-A concentration (data not shown). Even though it was suspected that cyclophilins may be possible partners of the *HeT-A* Gag protein, there is no evidence to support this hypothesis.

### Phosphorylation of *HeT*-A Gag at Serines 216 and 221 is important for protein stability

**Figure 5:**
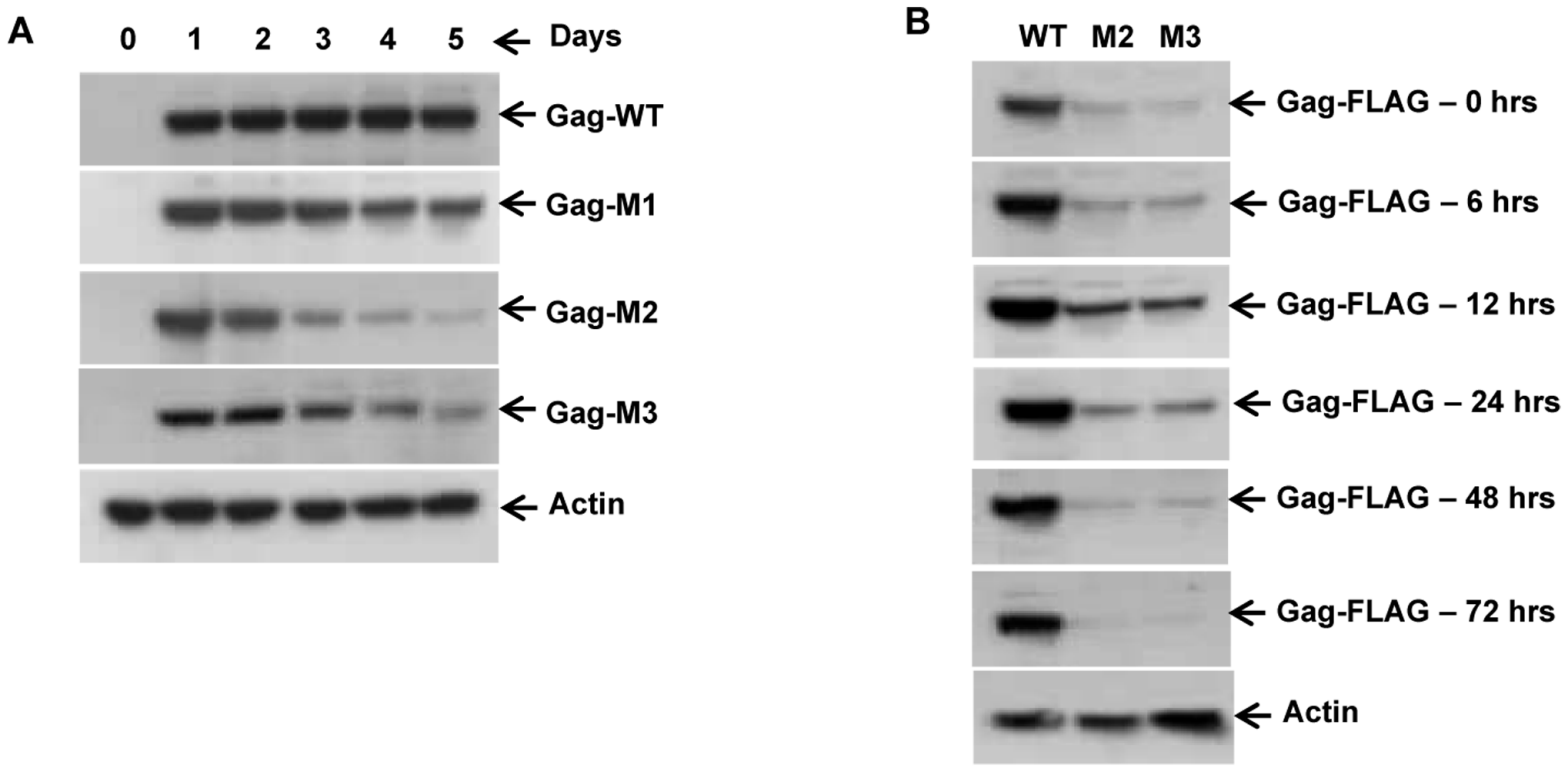
Time course of *HeT-A* Gag protein expression and proteasome treatment. (A) For the time course study, wild type, M1, M2, and M3 cells were seeded in T-25 flaks. At 80% confluence, cells were treated with 500 µM CuSO_4_, and equal numbers of cells were removed every 24 hrs for 5 days. Collected cells were lysed in SDS-PAGE buffer, subjected to immunoblotting, and the protein was detected by anti-FLAG antibody. Each membrane was stripped and probed for actin as a loading control. (B) For the proteasome treatment study, cells were seeded and induced as above. 72 hrs post CuSO_4_ inductions wild type, M2, and M3 cells were treated with 10 µM of proteasome inhibitor (MG132). Equal numbers of cells were harvested for various time points and analyzed by immunoblot.

To maintain cell integrity and survival, abnormal and misfolded proteins are selectively degraded by proteolysis [44]. Phosphorylation is one of the mechanism by which cellular proteins are marked for degradation, translocation, activation, or inactivation [45], [46]. To investigate if phosphorylation of serines 216 and 221 is important for *HeT-A* Gag protein stability, WT, M1, M2 and M3 *HeT-A* Gag stable cell lines were induced with 500 µM CuSO_4_. Cell fractions were collected every 24 hrs over a period of 5 days, total cellular proteins extracted using SDS-PAGE buffer, and analyzed by immunoblot. The level of *HeT-A* Gag protein in the M2 and M3 cell lines, compared to WT and M1, started decreasing from day 3 and continued to do so until the last point tested, day 5 (Fig. 5). To investigate if these double mutant proteins are being degraded by proteasomes, M2 and M3 stable cell lines where induced with 500 µM CuSO_4_, and 72 hrs post-induction cells were treated with 10 µM of the proteasome inhibitor MG132. Cells were removed at various times and analyzed by immunoblot (Fig. 5). At 12 hrs post MG132 treatment some protein accumulation was observed in both M2 and M3 cells, but later time points show the protein undergoing proteolysis, indicating that the recovery was only for a short period of time. Using higher concentration of proteasome inhibitor did not result in increased accumulation of protein (data not shown).

## Discussion

In most organisms telomeres are maintained by the enzyme telomerase [47], [48]. In *Drosophila*, telomerase is absent, and telomeres are maintained by targeted transposition of three non-LTR retrotransposons, *HeT-A*, *TART*, and *TAHRE*. These three elements are only found in telomeres, although tandem arrays of decayed fragments may be found in other heterochromatin regions. Among these three elements, *HeT-A* is the most frequent (90%) and thus has been widely studied. However, the exact mechanism by which *HeT-A* elements transpose to chromosome ends is still not known. The *HeT-A* element of *Drosophila* has one open reading frame that encodes for a Gag-like protein with homology to LINE Gag.

It is still not known how the *HeT-A* Gag-RNA-protein complex moves from the cytoplasm into the nucleus. Several possibilities can be proposed: First, if the protein is translated on the rough endoplasmic reticulum, it can move into the nucleus by lateral diffusion through a nuclear pore, since the outer nuclear membrane is in continuous with the endoplasmic reticulum. Second, if *HeT-A* Gag is synthesized in the cytoplasm, it can be transported to the nucleus through a nuclear pore complex [49]. Also, the *HeT-A* RNA-Gag protein complex translocation into the nucleus may depend upon the size of the complex; currently there are no data available which show how many Gag protein molecules bind per transcript. Additionally, translocation to the nucleus may be directed by interactions of *HeT-A* Gag with other proteins, such as cyclophilins. When we tested by blot analysis of different cellular fractions from a cyclosporine-A-treated stable cell line, we found that the *HeT-A* Gag protein was mostly present in the nuclear fraction. No changes in the *gag* expression profile were seen even at 100 µM cyclosporine-A concentration. While we can speculate from this observation that the *HeT-A* Gag protein may not be binding to cyclophilins, we understand this is not direct evidence and will need further investigation. However, this speculation is strengthened by the observation that Gag polyproteins of HIV-1 and closely related retroviruses (e. g. SIV-1) interact with the cyclophilin proteins, but the Gag proteins of more distantly related retroviruses (Mason-Pfizer Monkey Virus and Moloney murine leukemia virus) are not capable of carrying out this interaction [32]. This may be true with the *HeT-A* Gag protein as well. Nevertheless, once inside the nucleus the *HeT-A* Gag protein may be tightly bound to the inner nuclear membrane or to the nuclear matrix, as found for some TBPs. *HeT-A* Gag protein movement inside the nucleus might be tightly regulated because of their highly specialized role in genome stability. We can also speculate here that this tight association of *HeT-A* Gag might be related to a staging area inside the nucleus before these complexes are released and directed by some other protein(s) to the telomeres.

Our attempts to characterize the *HeT-A* Gag protein and to find its possible binding partners were hindered by our inability to dislodge it from nuclei under physiological conditions or after attempted solubilization by salts or detergents, although *HeT-A* Gag was readily degraded by Proteinase K. Future advances in methodology may provide more sound methods to break this strong interaction of *HeT-A* Gag with insoluble nuclear components. Protein phoshorylation has the potential to modulate protein-protein interactions and to alter the stability and/or subcellular localization of phospho-proteins. Here we show that specific phosphorylation of Ser 216 and Ser 221 in *Drosophila HeT-A* Gag had minimal effects on its subcellular localization, but is important for protein stability. To date ∼251 protein kinases have been identified in Drosophila [50]. Future investigation will determine which protein kinase/s phosphorylate *HeT-A* Gag, and the effects on transposition and chromosome stability. We believe that understanding the mechanism of transposition of retrotransposons is important not only to understand variant mechanisms of telomere maintenance, but also because 42% of the human genome consists of retrotransposons, and the mechanisms of their transposition are still not clear.

## Supporting Information

Figure S1 ***HeT-A*** **Gag-FLAG protein is resistant to different treatments.** (A) Nuclei from stable S2 cells expressing *HeT-A* Gag-FLAG protein were treated with 0.5 M NaCl, 1.0% Triton X-100, or 40 mM CHAPS. Supernatant (S) and pellet (P) fractions were separated and subjected to immunoblot analysis. In all 3 treatments almost all FLAG-tagged *HeT-A* Gag protein was detected in the P fractions. The same membranes were stripped and probed for actin and histone H1 as loading controls for cytoplasmic and nuclear fractions respectively. (B) Nuclei were treated with 0.5, 1.0, 1.5, or 2.0 M NaCl. Supernatant and pellet fractions were separated and subjected to immunoblotting. As seen from the blot, almost all *HeT-A* Gag-FLAG protein was present in the P fractions. Loading controls were detected as mentioned above. (C) Nuclei were treated with 0.01, 0.1, 1.0, or 2.0% SDS for 10 min at room temperature. Supernatant and pellet were separated and subjected to immunoblotting. (D) Nuclei were left untreated or treated with DNase, RNase-A, and DNase+RNase-A. (E) After completion of the reaction, an aliquot from each treatment was analyzed on an agarose gel (lane1 =  marker, lane 2 =  untreated, lane 3 =  DNase treated, lane 4 =  RNase treated, and lane 5 =  DNase+RNase treated) and the remaining reaction was spun to separate S and P fractions and analyzed by immunoblotting. (F) Nuclei were treated with benzonase nuclease for 30 min, or 1 or 2 hrs. (G) Part of the reaction was analyzed on an agarose gel (lane 1 =  marker, lane 2 =  untreated, lane 3 = 30 min, lane 4 = 1 hr, and lane 5 = 2 hrs) and the remaining reaction was spun to separate S and P fractions and analyzed by immunoblot. (H) Nuclei were treated with benzonase, NaCl, and SDS. S and P fractions were separated and analyzed by immunoblotting using an anti-FLAG antibody. (I-a) Nuceli were treated with various concentrations of Proteinase K for 15 min at room temperature. (I-b) Nuclei were treated for various length of time with Proteinase K (1 µg/10 ul nuclei extract).
(TIF)

Figure S2 **S2 cells expressing wild type** ***HeT-A*** **Gag-FLAG treated with cyclosporine-A.** Cytoplasmic and nuclear fractions, and nuclei were prepared as described in Materials and Methods and subjected to immunoblotting. Cyclosporine-A treatment did not block gag translocation to nucleus.
(TIF)
