# Supplementary figures and images for: Phosphorylation at Serines 216 and 221 Is Important for Drosophila HeT-A Gag Protein Stability
Source: PLoS One. 2013 Sep 18;8(9):e75381. doi: 10.1371/journal.pone.0075381 (PMC3776773; doi:10.1371/journal.pone.0075381)

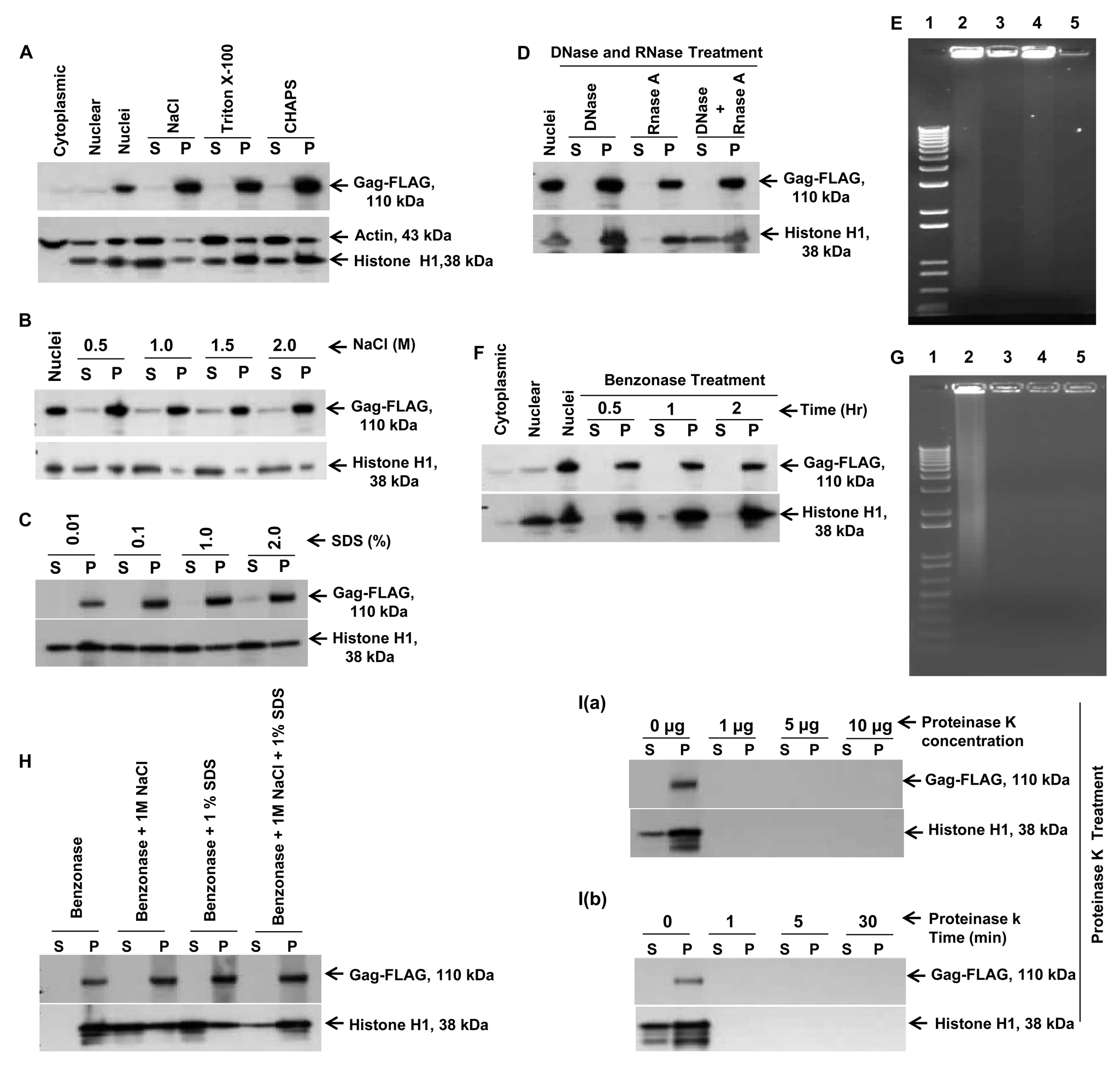

Supplement: Figure S1 — HeT-A Gag-FLAG protein is resistant to different treatments. (A) Nuclei from stable S2 cells expressing HeT-A Gag-FLAG protein were treated with 0.5 M NaCl, 1.0% Triton X-100, or 40 mM CHAPS. Supernatant (S) and pellet (P) fractions were separated and subjected to immunoblot analysis. In all 3 treatments almost all FLAG-tagged HeT-A Gag protein was detected in the P fractions. The same membranes were stripped and probed for actin and histone H1 as loading controls for cytoplasmic and nuclear fractions respectively. (B) Nuclei were treated with 0.5, 1.0, 1.5, or 2.0 M NaCl. Supernatant and pellet fractions were separated and subjected to immunoblotting. As seen from the blot, almost all HeT-A Gag-FLAG protein was present in the P fractions. Loading controls were detected as mentioned above. (C) Nuclei were treated with 0.01, 0.1, 1.0, or 2.0% SDS for 10 min at room temperature. Supernatant and pellet were separated and subjected to immunoblotting. (D) Nuclei were left untreated or treated with DNase, RNase-A, and DNase+RNase-A. (E) After completion of the reaction, an aliquot from each treatment was analyzed on an agarose gel (lane1 = marker, lane 2 = untreated, lane 3 = DNase treated, lane 4 = RNase treated, and lane 5 = DNase+RNase treated) and the remaining reaction was spun to separate S and P fractions and analyzed by immunoblotting. (F) Nuclei were treated with benzonase nuclease for 30 min, or 1 or 2 hrs. (G) Part of the reaction was analyzed on an agarose gel (lane 1 = marker, lane 2 = untreated, lane 3 = 30 min, lane 4 = 1 hr, and lane 5 = 2 hrs) and the remaining reaction was spun to separate S and P fractions and analyzed by immunoblot. (H) Nuclei were treated with benzonase, NaCl, and SDS. S and P fractions were separated and analyzed by immunoblotting using an anti-FLAG antibody. (I-a) Nuceli were treated with various concentrations of Proteinase K for 15 min at room temperature. (I-b) Nuclei were treated for various length of time with Proteinase K (1 µ [file pone.0075381.s001.tif]

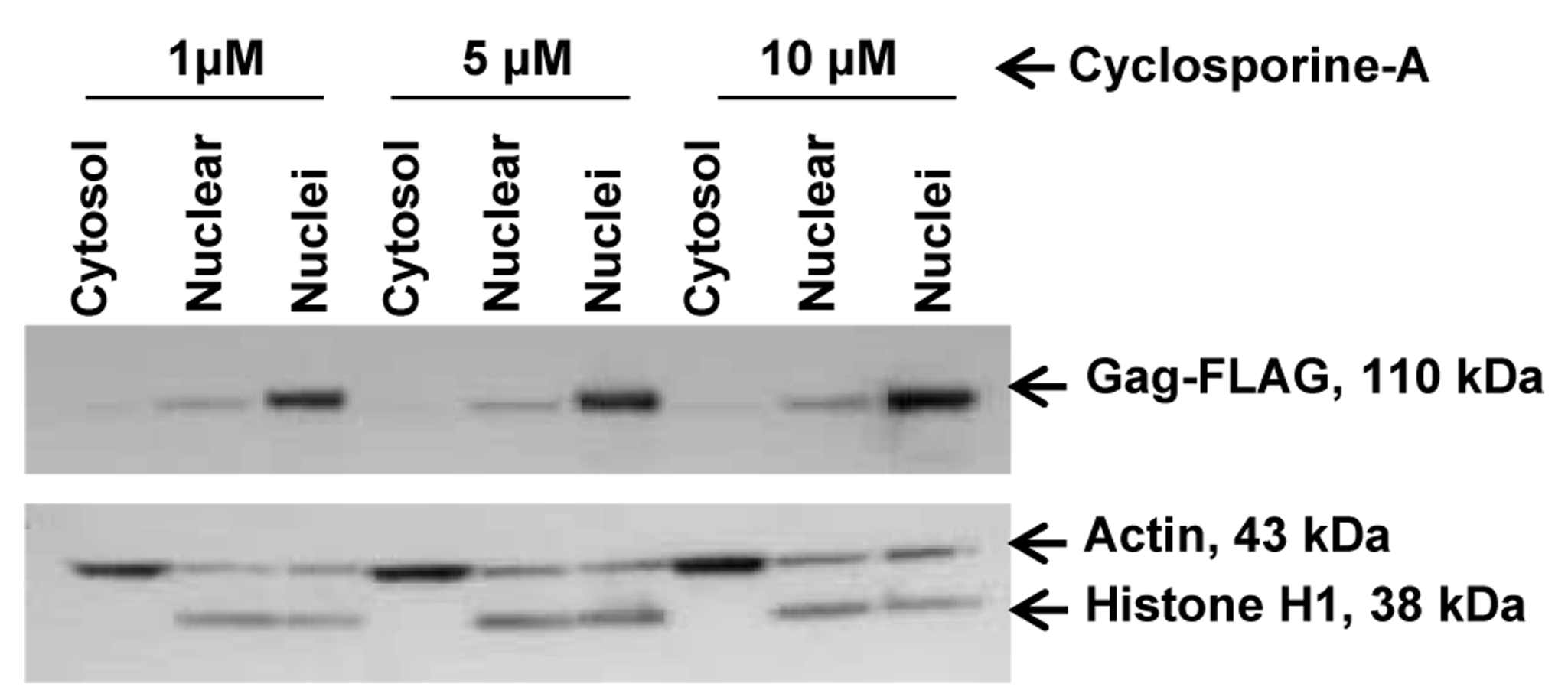

Supplement: Figure S2 — S2 cells expressing wild type HeT-A Gag-FLAG treated with cyclosporine-A. Cytoplasmic and nuclear fractions, and nuclei were prepared as described in Materials and Methods and subjected to immunoblotting. Cyclosporine-A treatment did not block gag translocation to nucleus. (TIF) [file pone.0075381.s002.tif]
